# Gpr176 modulates the firing pattern of parvalbumin-positive interneurons in the orbitofrontal cortex of mouse

**DOI:** 10.1186/s13041-025-01254-2

**Published:** 2025-11-04

**Authors:** Jing Tian, Ziran Huang, Wen Zhang

**Affiliations:** Department of Neurobiology, School of Basic Medical Sciences and National Institute on Drug Dependence, Peking University, 38 Xueyuan Road, Beijing, 100191 China

**Keywords:** Parvalbumin-positive interneuron, Action potential, Gpr176, The prefrontal cortex

## Abstract

Parvalbumin-positive (PV+) interneurons are the most abundant type of interneurons in the cortex. Its characteristic high-frequency non-accommodating firing pattern is critical for cortical inhibition, network activity, and mouse behavior. In the brain, neuromodulation via G protein-coupled receptors (GPCRs) regulates neuronal activities, including the output of neurons. GPCRs are the largest receptor superfamily, and there are GPCRs called “orphan GPCRs” whose endogenous ligands are still not clear. Meanwhile, studies have shown that some of them are constitutively active, but the modulation of these GPCRs on neuronal activity is far from clear. Among orphan GPCRs, Gpr176 is a constitutively active GPCR known for its role in the circadian rhythm in the central nervous system. In the present study, we found that the expression of Gpr176 was mainly expressed in PV + interneurons in the prefrontal cortex, and the knockdown of Gpr176 increased the output of PV + interneurons by increasing the membrane potential change in the repolarizing phase of action potentials in a train. We also found that the synaptic activities of these neurons were not affected. Furthermore, we observed changes in behaviors of mice with the knockdown of Gpr176 in the PV + interneurons of the prefrontal cortex. These data suggest an important role of Gpr176 in the regulation of intrinsic membrane properties of PV + interneurons in the prefrontal cortex.

**Supplementary Information:**

The online version contains supplementary material available at 10.1186/s13041-025-01254-2.

## Introduction

In the cortex, the inhibition is mostly mediated by GABAergic inhibitory interneurons. The GABAergic neurons show diverse function, anatomy, genetic, and biochemical properties, and based on these, cortical GABAergic neurons could be grouped into three major types, namely, Parvalbumin- (PV-), Somatostatin- (SST-), and Vasoactive Intestinal Peptide- (VIP-) positive interneurons [1]. Among these types of GABAergic neurons, PV + interneurons are mostly abundant, and they are critical in cortical inhibition [2]. For example, previous studies have shown that PV + interneurons unspecifically connect to all nearby pyramidal cells [3]; PV + interneurons also control the spatial–temporal dynamics of multineural activity by functionally sculpting neuronal ensembles [4]. Our studies also showed that in cortical microcircuit, PV + interneurons always exert an inhibitory role to the output of the microcircuit [5, 6]. Furthermore, changes in the function and activity of PV + interneurons underlie the symptoms of many brain diseases, such as schizophrenia [7], Rett syndrome [8], and anxiety and depression [9].

In the brain, neuronal activity is also subjected to neuromodulations, which include regulations by G protein-coupled receptors (GPCRs) [10–13]. GPCRs are the largest receptor superfamily, making up ~ 4% of the human genes [14] and nearly 13% of total membrane proteins [15]. GPCRs are important for body functions, ~ 30% of marketed pharmaceuticals target human GPCRs [16], especially in the treatment of psychological diseases, such as schizophrenia, anxiety, and depression [17, 18]. The superfamily includes at least 800 receptors that participate in diverse physiological and pathological functions, however, the endogenous ligands of some of GPCRs remain unidentified. These so-called orphan GPCRs account for ∼30% of the ∼400 non-olfactory human GPCRs, and the functions of these GPCRs are not well-illustrated [19]. On the other hand, recent studies show that they may have important functions in the central nervous system and other organs [20]. For example, studies have shown that the orphan GPCR Gpr176 in SCN is important for circadian rhythm [21, 22], and it also shows oncogenic roles in breast cancer and colorectal cancer [23, 24]. However, while studies have shown Gpr176 is constitutively active and expressed in the cortex [25, 26], the expression pattern and functional role of Gpr176 in cortical neurons are not clear.

Therefore, in the present study, we examined the expression of Gpr176 in neurons of the prefrontal cortex and evaluated its functional role in the activity of PV + interneurons and its possible contribution to mouse behavior.

## Methods

### Mice

Male mice aged 8–14 wks old were used. Mice strains includes C57BL/6 J (RRID:IMSR_JAX:000664) and *PV-Cre* (B6.Cg-*Pvalb*^*tm1.1(cre)Aibs*^/J, RRID:IMSR_JAX:012358). Mice genotyping was performed following the guidance of the Jackson Laboratory. Mice were maintained on a 12-h light/dark cycle with food and water *ad lib*. All procedures are in accordance with the National Institutes of Health *Guide for the Care and Use of Laboratory Animals*, and have been approved by Peking University Animal Care and Use Committee.

### Open field test (OFT)

Prior to testing, mice were transported to the test room and left undisturbed for at least 30 min. Animal activity in a plastic test arena (40 × 40 × 40 cm) was recorded with a camera mounted above the center. Mice were placed in the center of the arena for open field test. The activities of mice over 10 min were recorded. For chemogenetic modulation of behavior, clozapine N-oxide (CNO, 1 mg/kg, APExBIO, Cat # A3317) was *i.p.* injected 45 min before behavior test. 10 min of animal activities in the arena were analyzed with Matlab (MathWorks, RRID:SCR_001622). The central area was defined as the 30 × 30 cm square space in the middle of the arena. The variables measured were distance travelled, time in the perimeter and the central area.

### Surgery and virus injection

Adeno-associated viruses (AAV) were used for the present study, including rAAV-EF1α-DIO-EGFP-5′miR-30a-shGpr176 (Gol)-3′miR-30a, rAAV-EF1α-DIO-EGFP(BC-0015), rAAV-Ef1α-DIO-hM4D(Gi)-mCherry(BC-0155).(titers: ≥ 5.00 × 10^12^ GC/mL, BrainCase, China). The sequence of shRNA targeting *Gpr176* is GCTCGCTACTGGGAAACTTCA.

Viruses were bilaterally injected into the orbitofrontal cortex (OFC) of the mice aged 6–8 wks old. Mice were anesthetized with 2% isoflurane in oxygen at a flow rate of 2 L/min and mounted on a stereotaxic frame (RWD Instruments). Mouse body temperature was maintained with a heating pad at 37 °C. Sterile ocular lubricant ointment was applied to the corneas to prevent drying. The scalp fur was shaved, and the skin was cleaned with 70% alcohol and betadine. A hole was drilled at the injection site (A/P: +2.4 mm; M/L: 1.2 mm; DV –2.3 mm) using a 0.5-mm diameter round burr on a high-speed rotary micromotor (RWD Instruments). A total of 250 nL of virus solution was injected into each hemisphere at a rate of 50 nL/min using a micro pump and Micro4 controller (World Precision Instruments).

After the injection, the needle was kept in the location for 5–10 min before being slowly withdrawn. The hole was sealed with bone wax and the skin was sutured. Mice were returned to their home cage to recover from anesthesia in a 37 °C isothermal chamber.

14 days after virus injection, mice were subjected to experiments.

### Ex vivo electrophysiological recordings

Adult male mice aged 8–12 wks-old were anesthetized with isoflurane and decapitated. Brains were removed and sectioned in cold (0–4 °C) cutting solution (in mM): 87 NaCl, 3.0 KCl, 1.5 CaCl_2_, 1.3 MgSO_4_, 1.0 NaH_2_PO_4_, 26 NaHCO_3_, 20 D-glucose, and 75 sucrose, saturated with 95% O_2_ and 5% CO_2_ to obtain 250 μm-thick coronal sections with a vibratome (Leica VT1200S). Slices were transferred and incubated in a holding chamber containing (ACSF, in mM): 124 NaCl, 3.0 KCl, 2 CaCl_2_, 1.3 MgSO_4_, 1.0 NaH_2_PO_4_, 26 NaHCO_3_, and 2 D-glucose, saturated with 95% O_2_ and 5% CO_2_ at 33 °C for 30 min and then at room temperature for at least 30 min before recordings.

Parvalbumin-positive interneurons in the OFC were identified with fluorescence under the microscope (Olympus BX51WI). Brain slices were placed in a submersion type chamber continuously perfused with ACSF saturated with 95% O_2_ and 5% CO_2_ at 31–33 °C.

Spontaneous and miniature (with bath application of 1 μM TTX) EPSCs and IPSCs were recorded at -70 and 10 mV, respectively. The pipette solution contained (in mM): 110 cesium methanesulfonate, 15 CsCl, 4 ATP-Mg, 0.3 GTP, 0.5 EGTA, 10 HEPES, 4.0 QX-314 and 5.0 Na_2_-Phosphocreatine (pH 7.2, 270–280 mOsm with sucrose). The E/I ratio was defined as the ratio between the average total charges of sEPSC and sIPSC in 1 s.

For current-clamp recording, the pipette solution contained (in mM): 120 potassium gluconate, 10 KCl, 4 ATP-Mg, 0.3 GTP, 5.0 Na_2_-Phosphocreatine, 10 HEPES, and 2 EGTA (pH 7.2, 270–280 mOsm with sucrose).

To test the efficacy of DREADDs in regulating the excitability of infected neurons, CNO was bath-applied at a concentration of 10 µM.

Electrodes had resistances 2–4 MΩ. Series resistance was fully compensated using the bridge circuit of the amplifier MultiClamp 700B (Molecular Devices, RRID: SCR_018455). During experiments, the series resistance was constantly monitored. The series resistance was not compensated in voltage-clamp experiments. Data were discarded when series resistance was > 16 MΩ or change of series resistance was > 15%.

Recordings were made with Multiclamp 700B amplifier controlled by AxoGraph X (AxoGraph Scientific). Action potential threshold was estimated as the point when the slope of rising membrane potential exceeds 45 mV ms^−1^. Data were filtered at 4 kHz and digitized at 20 kHz. Data were analyzed offline with Axograph X and NeuroMatic [27] (RRID: SCR_004186) in Igor Pro (Wavemetrics, RRID: SCR_000325) for synaptic transmissions and Electrophys Feature Extraction Library [28] (v5.7.10) with Python (v3.13.0, RRID:SCR_008394) for intrinsic properties.

### Immunostaining and RNAscope® in situ hybridization assay

For immunostaining, mice were anesthetized with isoflurane, and then perfused with phosphate-buffer saline (PBS, pH 7.4) and then 4% paraformaldehyde (PFA) in PBS. Brain tissues were post-fixed with 4% PFA in PBS overnight at 4 °C, and 25-μm coronal sections were prepared with vibratome.

Immunostaining followed the standard protocols for free-floating sections. In brief, free-floating sections were incubated in blocking solution containing 3% normal donkey serum, 1% bovine serum albumin (BSA), and 0.3% Triton X-100 in PBS, with slow shaking for 2 h at 23–25 °C. Sections were then treated with primary antibody in blocking solution for overnight at 4 °C and with secondary antibody in blocking solution at 23–25 °C for 2 h with slow shaking. Primary antibodies used was Goat Anti-Parvalbumin (1:2000, Swant, Cat# PVG-213, RRID: AB_2650496). Secondary antibodies used was Alexa Fluor 546 Anti-Goat (1:250, Thermo Fisher Scientific, Cat# A11056, RRID: AB_2534103). We acquired fluorescent images with a confocal microscope (Leica TCS-SP8 DIVE) using a 10 × objective (NA/0.4).

To test the cell-type-specific expression of Gpr176 in the prefrontal cortex and AAV mediated cre dependent knockdown of Gpr176, we performed RNAscope in situ hybridization to quantify mRNAs of these molecules as described previously (5). Probes against the mRNAs of *Gpr176* (Cat#318,141), *Pvalb* (Cat# *421,931*), *Sst* (Cat# 404,631) and *Slc17a7*(Cat#501,101) were used. In brief, mouse brains were obtained and quickly frozen in isopentane on dry ice for 20 s. Coronal brain sections of 16 µm thickness were collected in a cryostat at − 20 °C. We next fixed those sections with 10% neutral buffered formalin (Millipore, Cat#HT5011), followed by washing steps with PBS and dehydrating steps with alcohol. With the RNAscope Multiplex Fluorescent Assay v2 (Advanced Cell Diagnostics, Cat#323100) and RNAscope 4-Plex Ancillary kit for Multiplex Fluor (Advanced Cell Diagnostics, Cat#323120), we treated sections with H_2_O_2_ for 10 min before with protease IV (Advanced Cell Diagnostics, RRID:SCR_012481) for 30 min in room temperature. After washing off the protease, we incubated brain sections with a mix of probe sets targeting mRNAs mentioned above for 2 h at 40 °C in the HybEZ oven (Advanced Cell Diagnostics). Following probe incubation, sections went through a series of incubations with preamplifier probes, amplifier probes, and TSA Vivid Fluorophore 570 (Advanced Cell Diagnostics, Cat#PG-323271) or TSA Vivid Fluorophore 650 (Advanced Cell Diagnostics, Cat#PG-323273) at 40 °C, and finally counter-stained with DAPI. We acquired fluorescent images with a confocal microscope (Leica TCS-SP8 STED) using a 63 × objective (NA/1.4). The maximal projection of a 5 µm thick stack was analyzed with ImageJ (ver. 1.53t) based FIJI (RRID:SCR_002285) [29]. The analysis was performed as previously described [6]. The combined region of *Slc17a7*/*Pvalb*/*Sst* and enclosed DAPI area was defined as the cell area. The fluorescent dots whose area were larger than 2 pixels were counted.

### Reagents

All reagents were from Sigma Aldrich.

### Sample size, randomization, and blinding statement

Sample sizes were estimated based on past experience and those presented in the literature. Typically, recordings of n > 10 neurons from at least three mice each group were collected for electrophysiological studies; n > 10 counts from each side of slices from at least three mice each group were collected for immunostaining and in situ hybridization; n = 6 for behavior tests. Mice were randomly allocated to treatment condition.

Initial behavior tests and electrophysiological recordings were performed in a blinded manner. All other data were collected and analyzed without the investigator blinded to genotype and treatment conditions.

### Statistical analysis

All reported sample numbers represent biological replicates. All statistical analyses and data plotting were performed with R (ver. 4.2.2, RRID: SCR_001905). The non-base attached packages for R were ggpubr (0.6.0), rstatix (0.7.2), tidyverse (2.0.0), emmeans (1.8.5). For boxplots, whiskers denoted 1.5 * IQR from the hinges, which corresponded to the first and third quartiles of distribution. For comparisons between two groups, non-parametric Wilcoxon test was used. For comparisons between multiple groups, one-way ANOVA with *post-hoc* Bonferroni test was used. For F-I analysis, two-way ANOVA with post hoc Games-Howell test was used. n, sample number of cells; N, sample number of mice. *p* < 0.05 is considered statistically significant.

### Data availability

The data that support the findings of this study are available from the corresponding author upon request.

## Results

**Fig. 1:**
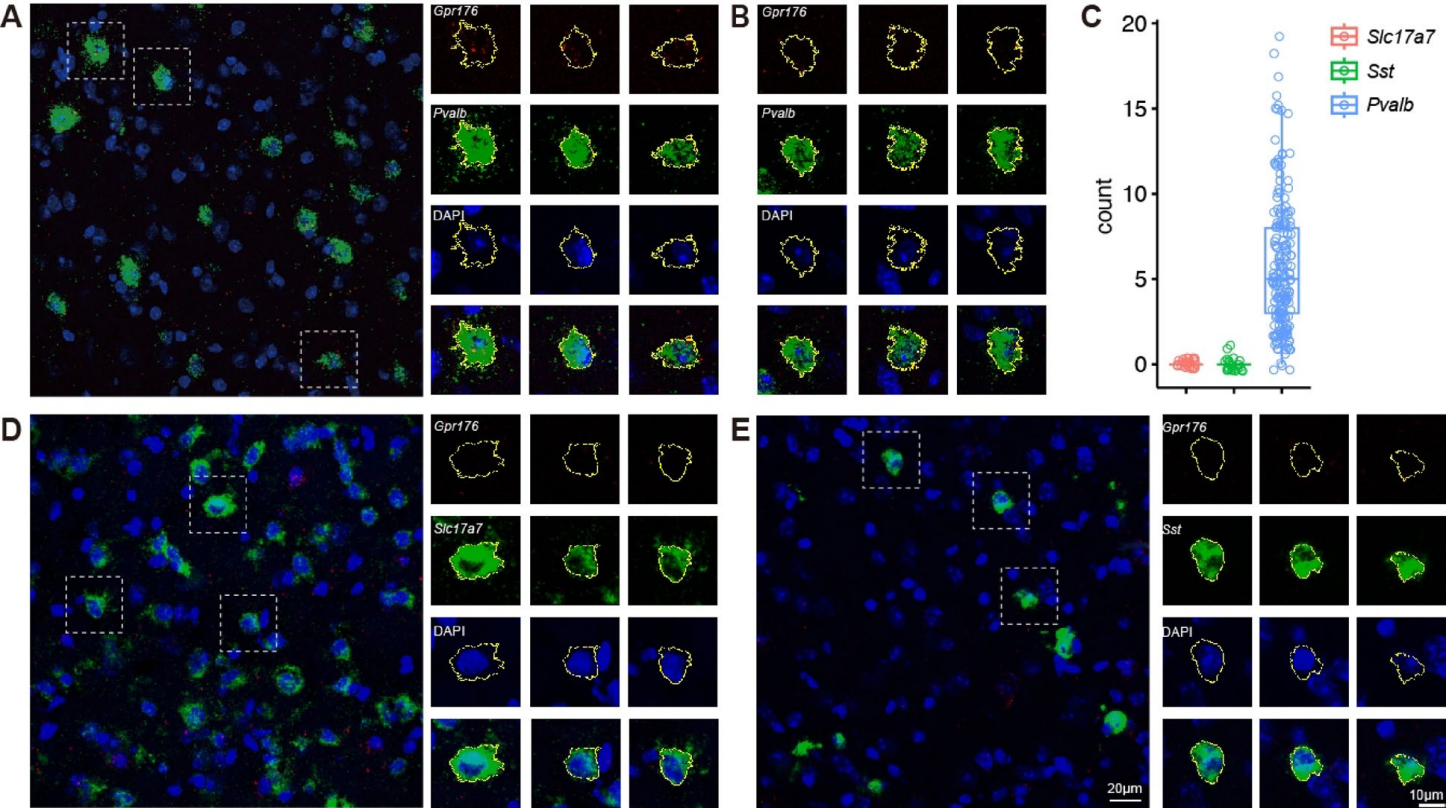
The cell-type-specific expression of *Gpr176* in the prefrontal cortex. RNAScope in situ hybridization of the mRNA of Gpr176 in the prefrontal cortex neurons, including *PV* + (*Pvalb* + , **A** and **B**), excitatory (*Slc17a7* + , **D**) and *SST* + (*Sst* +) (**E**) neurons. The closed yellow lines are the cell soma area defined by the combined region of *Slc17a7*/*Pvalb*/*Sst* and enclosed DAPI fluorescent signals. **C** statistical analysis of *Gpr176* puncta count per cells in *Slc17a7* + (n = 27), *SST* + (n = 23) and *PV* + (n = 191) neurons

To understand the role of Gpr176 in the prefrontal cortex, we first examined the expression of *Gpr176* mRNA in neurons of the orbitofrontal cortex (OFC), which is a part of the prefrontal cortex (Fig. 1). We found that *Gpr176* is primarily expressed in Parvalbumin-positive (PV +) cells, barely expressed in Somatostatin-positive (Sst +) cells or excitatory (Slc17a7 +) cells. (Fig. 1C; *Gpr176* puncta count per cell: Slc17a7 + , 0 ± 0, n = 27, N = 1; Sst + , 0.09 ± 0.06, n = 23, N = 1; PV + , 5.7 ± 0.28, n = 191, N = 3.)

**Fig. 2:**
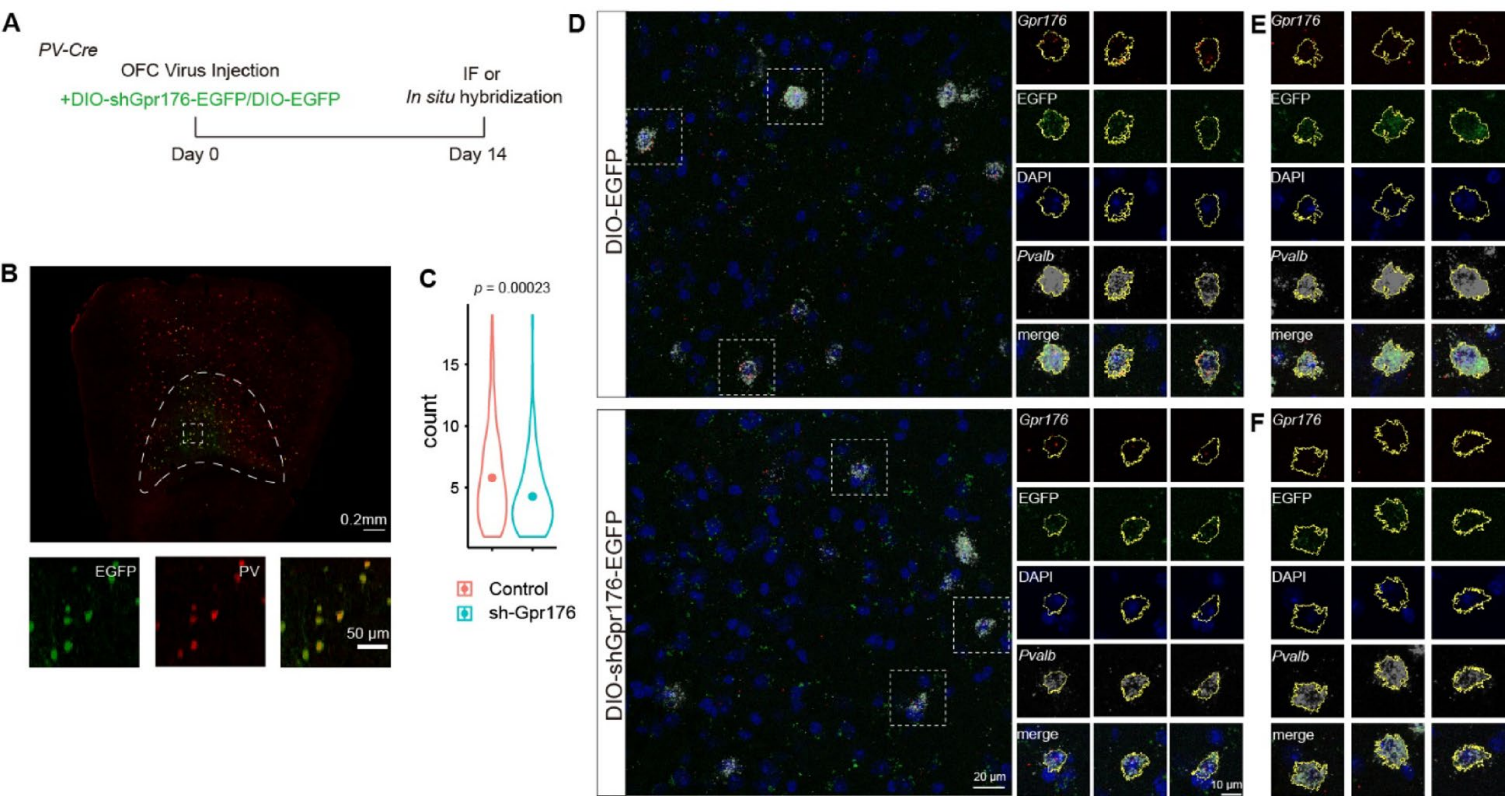
AAV mediated Cre dependent knockdown of Gpr176 reduced the mRNA of *Gpr176* in PV + interneurons. **A** Experiment timeline. **B** Top, representative viral infection in the OFC 2 weeks after virus injections. Bottom, blown-up view to show viral expression is located in PV + interneurons. **C** and **D** RNAscope in situ hybridization of Gpr176 in PV + interneurons of the OFC. **C** Representative image of control group (top) and sh-Gpr176 group (bottom). Right panels, blown-up view to show the expression of *Gpr176* of PV + interneurons of dashed square on image to the left. **D** Statistical comparison of *Gpr176* puncta count per cells of the control group and sh-Gpr176 group. **E** and **F** Additional images of the expression of the *Gpr176* mRNA in PV + interneurons of the control group (**E**) and sh-Gpr176 group (**F**). The closed yellow lines in (**D**–**F**) are the cell soma area defined by the combined region of *Pvalb* and enclosed DAPI fluorescent signals. Circles and bars in violin plots, mean ± sem. *p*, Wilcoxon test; Control, n = 186; sh-Gpr176, n = 129; N = 3/group

Gpr176 is an orphan GPCR, its endogenous ligand is still not identified [30]. To understand its role in PV + interneurons, we used the Cre-dependent AAV-mediated shRNA technique (AAV-DIO-shGpr176) to knockdown *Gpr176* in the OFC of PV-Cre mice (Fig. 2). Fourteen days after AAV-DIO-shGpr176 injected into the OFC of PV-Cre + mice, we first evaluated the expression of the *Gpr176* mRNA and found shGpr176 led to ~ 28% reduction of *Gpr176* mRNA expression in PV + interneurons (Fig. 2C; *Gpr176* puncta count per cell: Control, 5.80 ± 0.28, n = 186; sh-Gpr176, 4.28 ± 0.26, n = 129; N = 3/group).

**Fig. 3:**
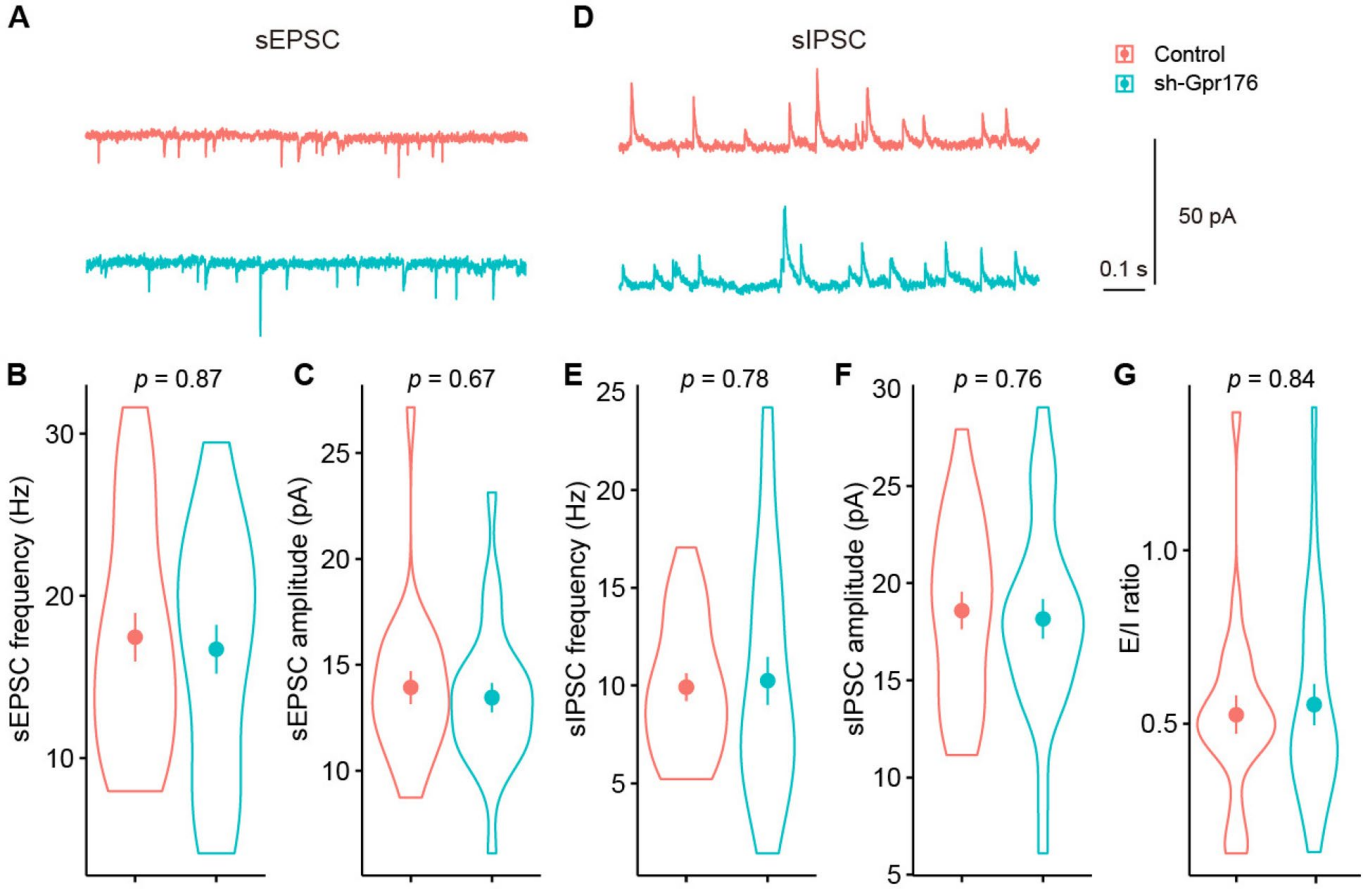
The knockdown of *Gpr176* did not change spontaneous excitatory and inhibitory transmission of PV + interneurons. **A**–**C** Representative traces and statistics of spontaneous excitatory postsynaptic currents (sEPSC) of PV + neurons of control and sh-Gpr176 mice. **D**–**F** Representative traces and statistics of spontaneous inhibitory postsynaptic currents (sIPSC) of PV + interneurons of control and sh-Gpr176 mice. **G** Comparisons of the excitation/inhibition ratio of PV + interneurons of the control and sh-Gpr176 mice. Circles and bars in violin plots, mean ± sem. *p*, Wilcoxon test; Control, n = 23, N = 7; sh-Gpr176, n = 25; N = 6

We then examined the effect of the knockdown of *Gpr176* on the activities of PV + interneurons (Fig. 3). We found knockdown of *Gpr176* change neither excitatory (Fig. 3B and C; sEPSC frequency [Hz]: Control, 17.40 ± 1.50; sh-Gpr176, 16.70 ± 1.50; sEPSC amplitude [pA]: Control, 13.9 ± 0.77; sh-Gpr176, 13.50 ± 0.68) nor inhibitory (Fig. 3E and F; sIPSC frequency [Hz]: Control, 9.91 ± 0.72; sh-Gpr176, 10.2 ± 1.22; sIPSC amplitude [pA]: Control, 18.6 ± 0.96; sh-Gpr176, 18.2 ± 1.02) spontaneous synaptic transmissions. The excitation/inhibition ratio of synaptic activities of PV + interneurons also did not change (Fig. 3G, E/I ratio: Control, 0.53 ± 0.06; sh-Gpr176, 0.56 ± 0.06. Control, n = 23, N = 7; sh-Gpr176, n = 25; N = 6).

**Fig. 4:**
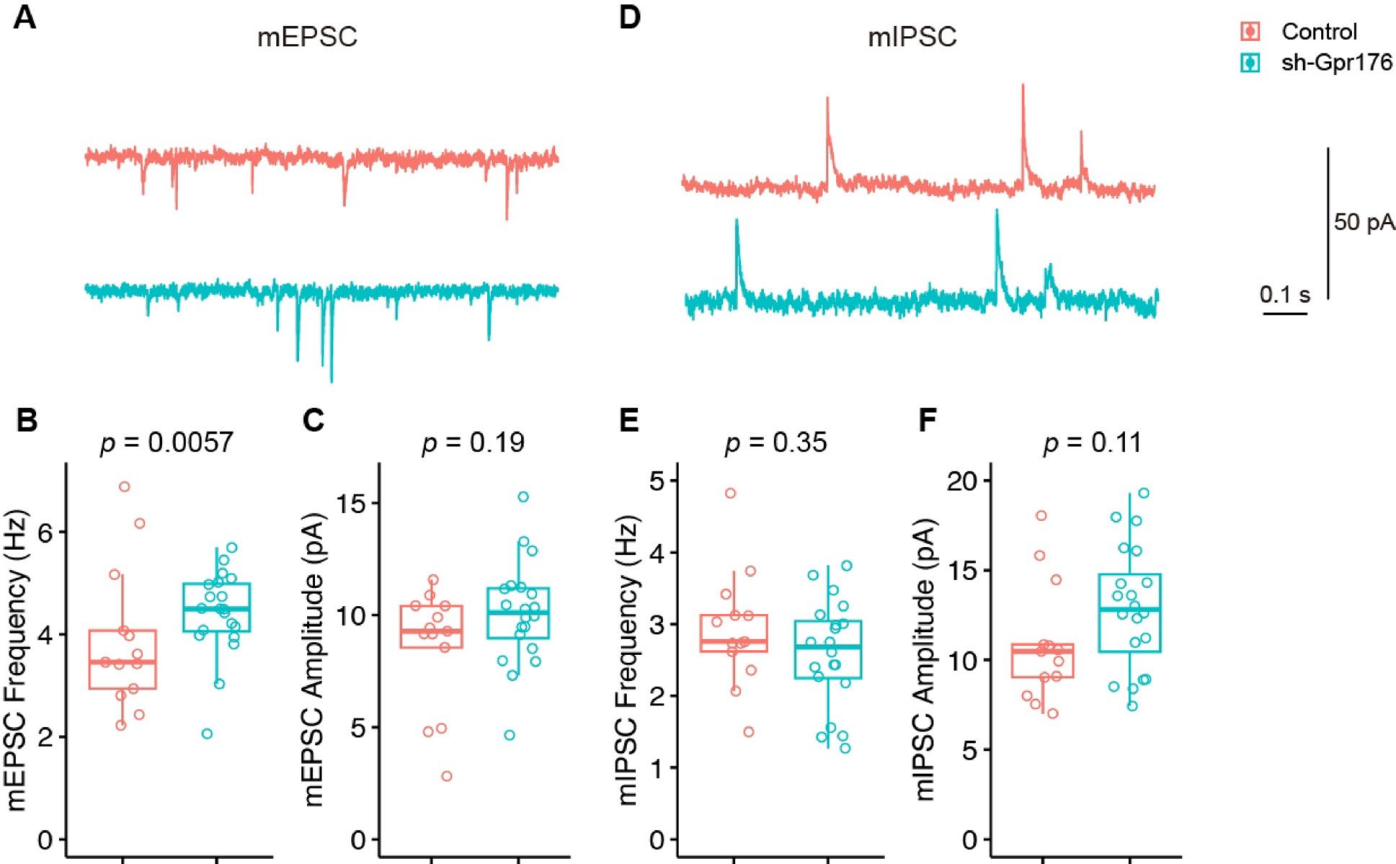
The knockdown of *Gpr176* did not change miniature excitatory and inhibitory transmission of PV + interneurons. **A**–**C** Representative traces and statistics of miniature excitatory postsynaptic currents (mEPSC) of PV + interneurons of control and sh-Gpr176 mice. **D**–**F** Representative traces and statistics of miniature inhibitory postsynaptic currents (mIPSC) of PV + interneurons of control and sh-Gpr176 mice. *p*, Wilcoxon test; Control, n = 13, N = 3; sh-Gpr176, n = 20; N = 4

The knockdown of *Gpr176* also did not change miniature synaptic transmissions of PV + interneurons (Fig. 4). Both the mEPSC (Fig. 4A–C; mEPSC frequency [Hz]: Control, 3.89 ± 0.39; sh-Gpr176, 4.40 ± 0.19; mEPSC amplitude [pA]: Control, 8.57 ± 0.74; sh-Gpr176, 10.10 ± 0.52) and mIPSC (Fig. 4D–E; mIPSC frequency [Hz]: Control, 2.92 ± 0.23; sh-Gpr176, 2.59 ± 0.17; mIPSC amplitude [pA]: Control, 10.90 ± 0.92; sh-Gpr176, 12.9 ± 0.77. Control, n = 13, N = 3; sh-Gpr176, n = 20; N = 4) were not affected.

**Fig. 5:**
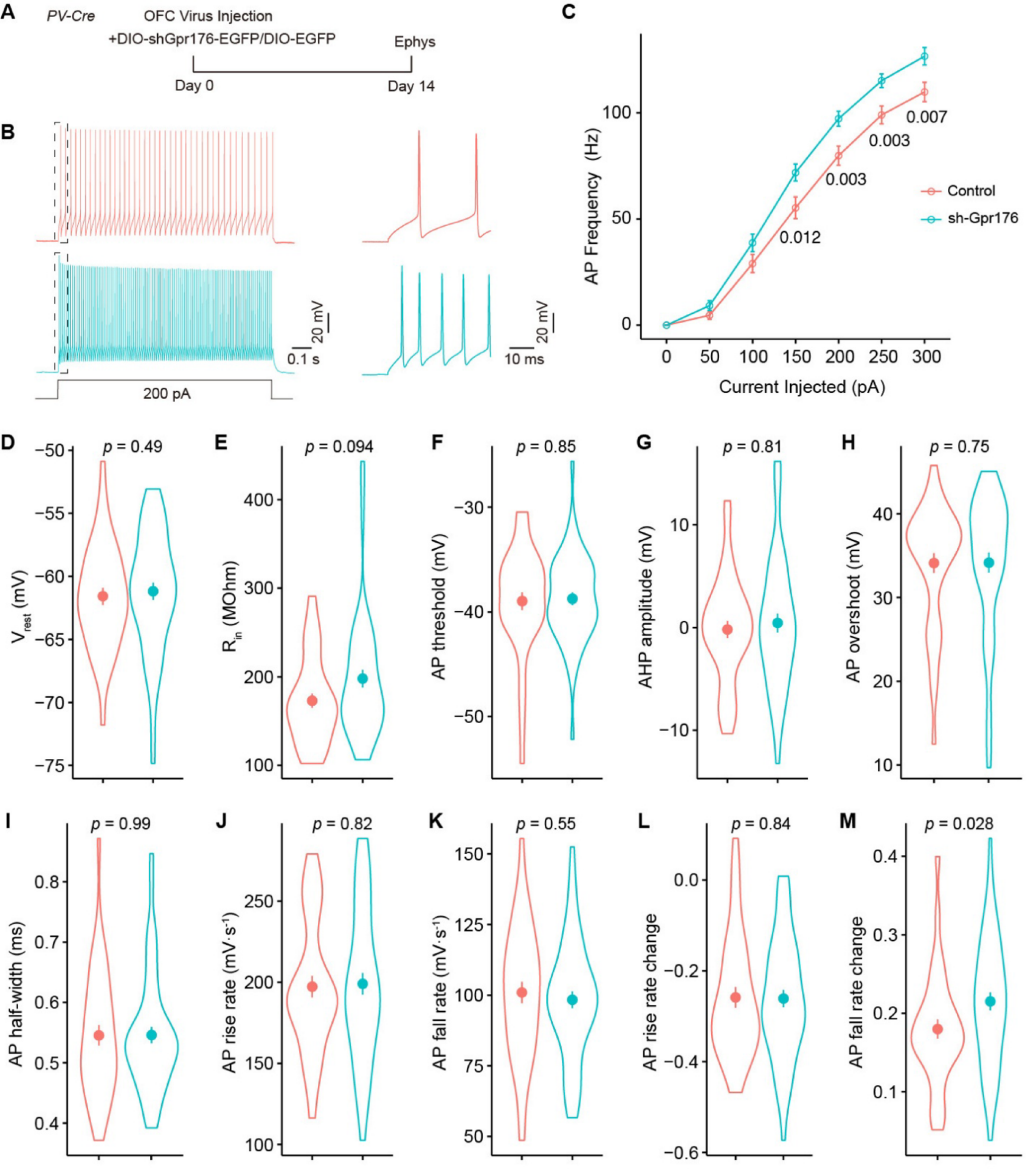
The knockdown of *Gpr176* increased the output of PV + interneurons. **A** Experiment timeline. **B** Representative traces of PV + interneurons spike evoked by 200 pA depolarization current. Blow-up views of the dashed rectangles are on the right. **C** F–I plot showed knockdown of *Gpr176* increased excitability of PV + interneurons. PV + interneurons evoked by a series of depolarization currents from 0 to 300 pA in 50 pA increments (two-way ANOVA, *p* = 0.004; *p* values computed with Games-Howell test presented on the plot). **D**–**M** Comparisons of the resting membrane potential (Vrest, **D**), input resistance (Rin, **E**), threshold of action potential (AP threshold, **F**), afterhyperpolarization depth of action potential (AHP amplitude, **G**), overshoot of action potential (AP overshoot, **H**), half-width of action potential (AP half-width, **I**), rise rate of action potential (AP rise rate, **J**), fall rate of action potential (AP fall rate, **K),** rise rate change of action potentials (AP rise rate change, **L**), and fall rate change of action potentials (AP fall rate change, **M**) of PV + interneurons of control and sh-Gpr176 mice. Circles and bars in violin plots, mean ± sem. *p*, Wilcoxon test; Control, n = 38, N = 5; sh- Gpr176, n = 50; N = 6

However, knockdown of *Gpr176* shifted the average firing frequency vs. current (F–I) relationships of PV + interneurons (Fig. 5A–C) while both the resting membrane potential (Fig. 5D; Control, − 61.60 ± 0.67 mV; sh-Gpr176, − 61.20 ± 0.68 mV) and input resistance (Fig. 5E, Control, 173.00 ± 8.02 MOhm; sh-Gpr176, 198.00 ± 9.99 MOhM) of PV + interneurons did not change. The kinetics of action potentials (AP) of the PV + interneurons evoked by current injection were also not affected by the knockdown of *Gpr176* (Fig. 5F–K; AP threshold [mV]: Control, − 39.00 ± 0.84; sh-Gpr176, − 38.80 ± 0.60; AHP amplitude [mV]: Control, − 0.19 ± 0.84; sh-Gpr176, 0.45 ± 0.91; AP overshoot [mV]: Control, 34.60 ± 1.19; sh-Gpr176, 34.70 ± 1.20; AP half-width [ms]: Control, 0.55 ± 0.02; sh-Gpr176, 0.55 ± 0.01; AP rise rate [mV/s]: Control, 197.00 ± 6.64; sh-Gpr176, 199.00 ± 6.58; AP fall rate [mV/s]: Control, 101.00 ± 3.76; sh-Gpr176, 98.40 ± 2.99).

We then compared both the rise rate change and fall rate change of APs evoked by the injection of 200 pA current (Fig. 5L and M; AP rise rate change: Control, − 0.26 ± 0.02; sh-Gpr176, − 0.26 ± 0.02; AP fall rate change: Control, 0.18 ± 0.01; sh-Gpr176, 0.22 ± 0.01. Control, n = 38, N = 5; sh- Gpr176, n = 50; N = 6). Interestingly, knockdown of Gpr176 only affected the change in the fall rate of action potentials in a train, *i.e.,* in a train of action potentials the repolarizing phase of action potentials accelerated. Combined with these results, the knockdown of *Gpr176* might change the repolarization of action potentials in a high-frequency train, which then led to the increased firing capability of these neurons.

**Fig. 6:**
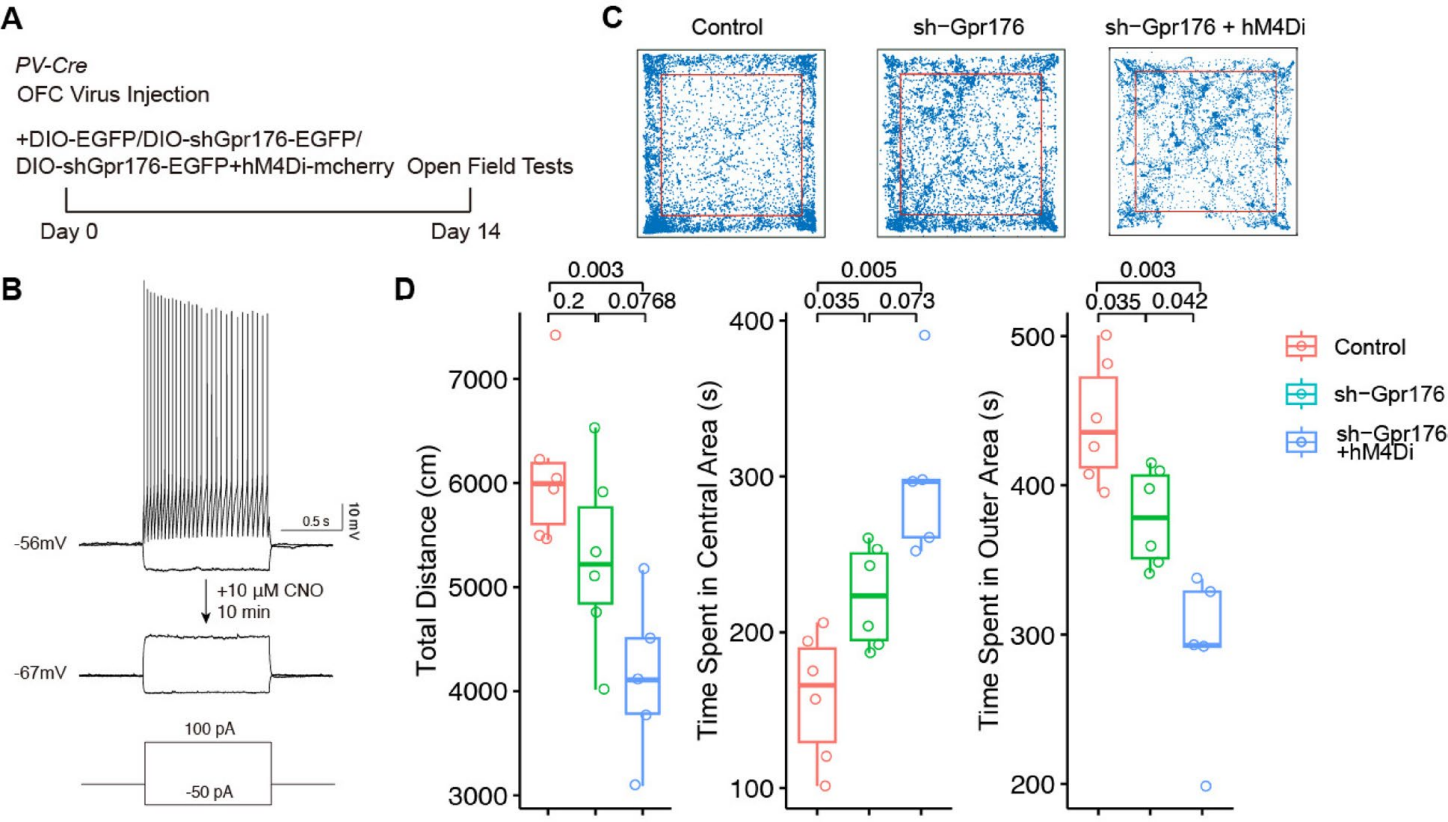
The knockdown of *Gpr176* in PV + interneurons led to decreased anxiety-like behavior of mice. **A** Experiment timeline. **B** Validation of the chemogenetic inactivation of PV + interneurons by 10 µm CNO. **C** and **D** Open field test of mice 2 weeks after viral injection. **C** Representative traces of mice in open field arena for 10 min with red squares denote the central area. **D** Statistical comparisons of the distance mice travelled in the arena (left; one-way ANOVA, *p* = 0.0004; *p* value computed with *post-hoc* Bonferroni test presented on the plot), time in the central area (middle; one-way ANOVA, *p* = 0.000385; *p* value computed with *post-hoc* Bonferroni test presented on the plot), and time in the perimeter area (right; one-way ANOVA, *p* = 0.000171; *p* value computed with *post-hoc* Bonferroni test presented on the plot) for 10 min. Control, N = 6; sh-Gpr176, N = 6; sh-Gpr176 + hM4Di, N = 5

Finally, we evaluated whether the knockdown of *Gpr176* in the PV + interneurons of the OFC could affect animal behavior (Fig. 6). We analyzed animal behavior in an open filed arena and found that while knockdown of *Gpr176* did not change the locomotor activity of mice (Fig. 6D; Total distance [cm]: Control, 6096 ± 292; sh-Gpr176, 5275 ± 359), the dwell time in the central area of the arena increased compared with the control (Fig. 6D; Time spent in central area [s]: Control, 159.0 ± 16.9; sh-Gpr176, 223.0 ± 13.3; Time spent in outer area [s]: Control, 443.0 ± 16.9; sh-Gpr176, 378.0 ± 13.3), suggesting decreased anxiety-like behavior. As knockdown of *Gpr176* increased the excitability of PV + interneurons, we also evaluated whether suppressed PV + interneuron activity with chemogenetic inhibition would restore affected behavior by the knockdown of *Gpr176*. We injected Cre-dependent shGpr176 together with Cre-dependent hM4Di into the OFC of PV-Cre mice (sh-Gpr176 + hM4Di). We found that 10 µm of CNO could block evoked action potentials of PV + interneurons (Fig. 6C). Interestingly, blockage of PV + interneurons in vivo with 1 mg/kg CNO compromised locomotion of these mice (Fig. 6D; Total distance: sh-Gpr176 + hM4Di, 4129 ± 347 cm). Meanwhile, mice injected with sh-Gpr176 + hM4Di showed higher dwelling time in the central area (Fig. 6D; Time spent in central area: sh-Gpr176 + hM4Di, 300.0 ± 24.6 s; Time spent in outer area: sh-Gpr176 + hM4Di, 290 ± 24.6 s).

## Discussion

In the present study, we showed that Gpr176 majorly expressed in PV + interneurons in the orbitofrontal cortex, and knockdown of Gpr176 in PV + interneurons increased output of these neurons while synaptic activities of which did not change. Furthermore, the knockdown of Gpr176 in PV + interneurons of the prefrontal cortex led to decreased anxiety-like behavior in mice.

Previous studies on Gpr176 have shown that it is a Gz-linked orphan GPCR [21]. It exhibits constitutive, agonist-independent activity that leads to reduced cAMP synthesis [31, 32]. In the central nervous system, it is enriched in the suprachiasmatic nucleus (SCN) [21] but also expressed in other brain regions, such as the prefrontal cortex, the dorsal raphe nucleus, and the cerebellum [25, 33]. Studies have shown that knockout of Gpr176 changed the circadian rhythm of mice, indicating disturbed neuronal function after Gpr176 knockout. In this study, we focused on whether the knockdown of Gpr176 could modulate neuronal function and found that knockdown of Gpr176 facilitated the output of PV + interneurons.

Parvalbumin-positive (PV +) interneurons is a major type of inhibitory neuron, and it is the largest interneuron population in the brain [1, 2]. They are characterized by the high-frequency firing upon activation, which is fundamental for fast and precise inhibition of pyramidal cells. This unique property of PV + interneurons among interneurons is postulated as coincidence detectors as a pyramidal cell will need multiple coincident inputs to overcome PV + interneurons inhibition and fire an action potential [34]. Besides, in the cortex, PV + interneurons are critical for the control of neuronal networks [35–39], the regulation of animal behaviors [40, 41], and malfunctions of these neurons contributed to several brain disorders [5, 6]. PV + interneurons are well-known for the high-frequency firing, which is non-accommodating [42], and studies showed that this firing pattern of PV-INs is mediated by both sodium and potassium channels [42–44]. In the present study, we found that the knockdown of Gpr176 only affected the change in the fall rate of action potentials in a train of spikes, indicating a possible change in the activity of potassium channels after Gpr176 knockdown. In cortical PV + interneurons, Kv3 and Kv1 subfamilies of voltage-gated potassium channels are well-known for their role in high-frequency firing of the neurons [45–47]. Studies suggest that GPCRs could modulate both Kv3 and Kv1 subfamily potassium channels [48, 49]. However, it is not clear which types of potassium channels of PV + interneurons are subjected to the modulation by Gpr176 activity.

A previous study has shown that the densities of PV + interneurons in the OFC were inversely correlated with anxiety levels of adult mice [50]. Considering the high firing frequency of PV + interneurons discharged (> 20 Hz) in vivo [51, 52], such correlation indicates increasing the firing capability of PV + interneurons in the OFC could reduce the anxiety levels of mice. Indeed, in the present study, we found knockdown of *Gpr176* led to decreased anxiety of mice in the OFT test. Interestingly, blockage of PV + interneuron activity with chemogenetic modulation reduced locomotion of mice in the OFT test. Accompanied such change, mice with PV + interneuron activity blocked also showed a significant change in the dwelling time in the central area of the test arena. However, considering the mice were placed in the central area at the beginning of the OFT test, it is not clear whether such a change in the dwelling time in the central area is due to changes in locomotion ability or the anxiety levels of these mice after chemogenetic modulation of PV + interneuron activity.

In summary, Gpr176 is a PV + interneuron-specific orphan GPCR in the orbitofrontal cortex. A deficiency of Gpr176 in PV + interneurons increased its output, leading to a higher frequency of firing. Furthermore, we did not observe change in the synaptic transmissions, including both excitatory and inhibitory of those PV + interneurons with reduced Gpr176 expression. The increased activity of PV + interneurons after the knockdown of Gpr176 suggests enhanced inhibitory activity in the orbitofrontal cortex of affected mice, and affected mice showed lower levels of anxiety-like behavior. Knockdown Gpr176 of PV + interneurons and inhibited their activities at the same time did not restore the anxiety level, which further suggests that Gpr176 in PV + interneurons is critical to brain functions.

## Supplementary Information

Below is the link to the electronic supplementary material.

Supplementary Material 1

## Data Availability

The data that support the findings of this study are available from the corresponding author upon request.
